# Recovery capital among people receiving treatment for opioid use disorder with buprenorphine

**DOI:** 10.1186/s12954-021-00553-w

**Published:** 2021-10-13

**Authors:** Anna Beth Parlier-Ahmad, Mishka Terplan, Dace S. Svikis, Laura Ellis, Caitlin E. Martin

**Affiliations:** 1 Department of Psychology, Virginia Commonwealth University, 401 N. 11th Street, Richmond, VA 23219 USA; 2 Friends Research Institute, Baltimore, MD USA; 3 Virginia Commonwealth University School of Medicine, Virginia Commonwealth University, Richmond, USA; 4 Department of Obstetrics and Gynecology and Institute for Drug and Alcohol Studies, Virginia Commonwealth University, Richmond, USA

**Keywords:** Recovery, Recovery capital, Opioid use disorder (OUD), Buprenorphine, Medication for opioid use disorder (MOUD)

## Abstract

**Background:**

Recovery is a multidimensional process that includes health, quality of life, and citizenship. Recovery capital is a strengths-based concept representing the sum of an individual’s resources that support recovery. This study (1) describes recovery capital, (2) examines the relationship between recovery capital and treatment duration, and (3) assesses differences by gender in recovery capital among people receiving medication for opioid use disorder (MOUD).

**Methods:**

This is a secondary data analysis of a cross-sectional study, with survey and medical record review components, conducted with patients recruited from an office-based opioid treatment clinic between July and September 2019. Analyses included participants receiving MOUD with buprenorphine who completed the Brief Assessment of Recovery Capital (BARC-10; *n* = 130). Univariate analyses explored differences by gender. Multivariate linear regression assessed the relationship between BARC-10 total score and length of current treatment episode.

**Results:**

Participants were 54.6% women and 67.4% Black with mean age of 42.4 years (SD = 12.3). Mean length of current MOUD treatment was 396.1 days (SD = 245.9). Total BARC-10 scores were high, but participants perceived low community-level resources. Women scored higher than men within the health and purpose recovery dimensions. While length of treatment was not associated with BARC-10 score, experiencing recent discrimination was associated with a significantly lower BARC-10 score.

**Conclusions:**

Recovery capital among individuals receiving MOUD was high suggesting that participants have resources to support recovery, but gender differences and prevalent discrimination highlight areas for improved intervention. More work is needed to investigate recovery capital as an alternative treatment outcome to abstinence in outpatient MOUD populations.

## Background

Historically, substance use disorder (SUD) treatment has focused on acute stabilization and achieving abstinence rather than long-term holistic recovery [1]. Unfortunately, within this care model, treatment gains tend to be short lived, and rates of substance use recurrence are substantial [1, 2]. Given our contemporary understanding of addiction as a complex chronic disease with a neurobiological basis modified by one’s environment and experiences, focusing on elimination of drug or alcohol use is an insufficient goal of SUD treatment [1–5]. The current opioid crisis, persistent increasing drug overdose rates [6], and expansion of treatment utilizing medication for opioid use disorder (MOUD) further underscore the need to reconceptualize treatment goals [7]. MOUD is a unique form of SUD treatment in that it requires ongoing care with a healthcare provider and long-term engagement. However, the primary data routinely collected to track MOUD progress and outcomes are urine drug test results, data that solely measure recent substance use.

Compared to non-pharmacological therapies, MOUD is consistently associated with better treatment retention rates and reduced substance use [8–10]. Yet treatment outcomes vary widely among those receiving MOUD highlighting substantial opportunities to improve treatment quality. For example, individuals with more stress, low mood, and higher levels of cravings are more likely to discontinue treatment [11]. Identifying and targeting non-abstinence-based outcomes is critical to advance MOUD treatments within the chronic disease model and reduce the adverse medical, public health, and social consequences associated with opioid epidemic.

Alternative treatment goals can be gleaned from studies of individuals with SUDs who are not in specialized treatment. The vast majority of individuals with SUD are not in specialized SUD treatment (12), yet many are able to initiate and maintain recovery [13, 14]. Prior research among this large group has explored resources beyond simply abstinence that support long-term holistic recovery such as improved health, quality of life, and citizenship [15–17]. Recovery capital is a strengths-based concept that refers to the sum of an individual’s resources that can be drawn upon to initiate and maintain recovery from addiction [18]. Factors that impede recovery, such as poor mental health, incarceration, stigma or discrimination, are conceptualized as negative recovery capital [19, 20]. In line with the chronic nature of addiction, recovery capital can be continually accumulated or exhausted throughout one’s life [20]. Prior work among nontreatment populations suggests high levels of recovery capital predict sustained abstinence, improved quality of life, and lower stress [13]. Further, recovery capital tends to increase with longer periods of remission [13, 16]. Less is known about recovery capital from a harm reduction perspective.

Guided by SUD treatment governing bodies, MOUD settings have begun to shift from the abstinence-based definition of treatment success to a more holistic, patient-centered view of recovery. In 2019, the Substance Abuse and Mental Health Services Administration (SAMHSA) developed a clinically oriented recovery framework including a working definition of recovery—“*a process of change through which individuals improve their health and wellness, live a self-directed life, and strive to reach their full potential”—*to help improve recovery-oriented systems of care and better meet the needs of those receiving SUD treatment regardless of substance use status [21]. SAMHSA also highlighted four domains that support recovery within their framework including (1) health—overcoming or managing disease and making healthy choices to support wellbeing, (2) home—having a stable and safe place to live, (3) community—building supportive relationships and social networks, and (4) purpose—engaging in meaningful daily activities as well as having the independence and resources to participate in society.

In addition to the paradigm shift in the concept of recovery, the Director of the National Institute on Drug Abuse has called for more personalized SUD treatment regimens in which resources provided to patients are individualized to meet their needs with a focus on optimizing recovery from a holistic perspective [4]. The standard of care for opioid use disorder (OUD) treatment combines medication (e.g., buprenorphine or methadone) with wrap-around services including medical care, mental health counseling, case management, and recovery support [22]. Assessing and targeting recovery capital during MOUD treatment could help promote a strengths-based approach to personalized SUD treatment and improve holistic recovery outcomes, such as quality of life, without necessarily requiring reduced substance use.

Few studies have explored recovery capital within SUD treatment settings. One recent study among patients in SUD treatment found that higher levels of recovery capital were predictive of SUD treatment completion [23]. Another study found that women and men in treatment for OUD qualitatively describe different resources used to sustain recovery [24]. Specifically, women describe having more social and material support (e.g., housing) than men. However, women also highlight health issues associated with domestic violence, reproduction, self-harming, and suicide behaviors as well as stigma as undermining their recovery efforts. To date, recovery capital among patients in outpatient MOUD treatment has not been reported.

As the number of office-based opioid treatment programs and buprenorphine prescribing providers continue to increase in response to the opioid crisis, more clinically relevant data focused on improving our understanding of recovery capital from a harm reduction perspective among men and women engaging in MOUD are needed to guide recovery-based systems of care [7, 10]. The primary objectives of this study are to: 1) describe recovery capital among an outpatient clinical sample receiving MOUD and 2) examine the relationship between recovery capital and length of time in treatment. We hypothesize that MOUD could be a modality to help improve recovery capital through activation of previously acquired strengths and development of new assets leading to better recovery outcomes. The secondary objective of this study is to explore gender differences in recovery capital as we hypothesize that men and women receiving MOUD will endorse different types of recovery capital.

## Methods

### Design

This is a secondary data analysis of a cross-sectional study with both survey and medical record review components. The parent study recruited a convenience sample of English-speaking, adult patients (*n* = 162; 97% response rate) from an outpatient addiction medicine clinic to complete a voluntary, electronic survey between July and September 2019 assessing reproductive and sexual health needs. Patients were recruited through flyers and referrals by clinic staff. All participants provided verbal consent. Those who were unable to read could have the survey read aloud by a research assistant in a private space (*n* = 6). Survey completion took an average of 40 min, and participants were compensated $20. A retrospective medical record chart abstraction was also conducted for parent study participants. Virginia Commonwealth University’s Institutional Review Board approved the study.

### Setting and participants

The office-based opioid treatment center provides outpatient addiction services for over 500 adults with the majority receiving buprenorphine. It is affiliated with a large academic medical center in a Medicaid-expanded state which serves as a safety net for the region and treats predominately individuals with low incomes and identifying as a racial or ethnic minority. On-site addiction medicine providers come from multiple specialties, including psychiatry, internal medicine, family medicine, obstetrics and gynecology, and emergency medicine. Most patients are referred from within the academic medical center (e.g., inpatient consults, primary care physicians). At the initial clinic visit, providers complete a comprehensive intake assessment. All patients diagnosed with OUD are offered buprenorphine. Patients typically have follow-up visits at least once every four weeks. A comprehensive, recovery-oriented care model is utilized in which patients have access to integrated on-site psychiatric, mental health, case management, and social work services. The clinic prioritizes a low threshold, harm reduction approach whenever possible, meaning that established patients with recurrence of substance use are not initially exited from treatment but instead first provided with increased wrap-around support.

The present secondary analytic study inclusion criteria were 1) receipt of MOUD with buprenorphine at the time of the survey and 2) completion of the outcome measure—Brief Assessment of Recovery Capital (BARC-10). Analyses included *n* = 130 participants (59 cisgender men and 71 cisgender women).

### Recovery capital

The primary outcome was recovery capital measured by the 10-item Brief Assessment of Recovery Capital (BARC-10) which is an abbreviated version of the original 50-item Assessment of Recovery Capital (ARC) with 10 subscales [25]. The BARC-10 retained and combined one item from each of the original 10 subscales from the ARC (substance use and sobriety, global psychological health, global physical health, community involvement, social support, meaningful activities, housing and safety, risk-taking, coping and life functioning, and recovery experience) to represent recovery capital [26], and its brevity provides more utility in clinical settings such as where this study was conducted. The BARC-10 uses a 6-point agreement scale [1-strongly disagree to 6-strongly agree] in response to the following instruction, “please respond to each statement based on how you are feeling today.” Item scores are summed for a total score ranging from 10 to 60 with higher scores indicating more recovery capital. For the current study, the primary recovery capital outcome was measured by the total score on the BARC-10. Prior research among a US sample of nontreatment seeking individuals with a resolved drug or alcohol problem found average BARC-10 scores were approximately 43 (SD = 10) [16]. Additionally, we used SAMSHA’s recovery framework to categorize and describe findings from the individual BARC-10 items within a clinically oriented context [21]. BARC-10 items were categorized into SAMHSA’s recovery dimensions as follows: 1) *health*—included the substance use and sobriety, global psychological health, and global physical health items (α = 0.73); 2) *home*—included the housing and safety item; 3) *purpose*—included the meaningful activities, coping/life functioning, risk-taking, and recovery experience items (α = 0.81); and 4) *community*—included the community involvement and social support items (α = 0.73).

### Demographic, psychosocial and clinical variables

Survey demographic items included gender (cisgender man, cisgender woman, transgender man, transgender woman, other), age, race, employment, education, marital status, and living arrangement. Psychosocial items were asked in reference to the past 12 months. Homelessness (yes/no) was defined as living on the street, in a shelter, in a single room occupancy hotel, or in a car. Food insecurity (yes/no) was assessed using an item from the Health Leads Social Determinants of Health Screening Tool [27]. Recent discrimination in healthcare settings for substance use (yes/no) was assessed by the following question: “In the past 12 months, have you ever felt you were treated unfairly getting health care services because of your drug and/or alcohol use?” For psychiatric comorbidity (yes/no), one item asked: “Have you ever had or been treated for depression, anxiety, post-traumatic stress disorder, or any other psychiatric condition?” Healthcare access (yes/no) was defined as seeing a provider other than an addiction provider at least once in past 12 months. The Medical Outcomes Study Social Support Survey measured social support using a 6-point frequency scale [0-none of the time to 5-all of the time]; higher average scores indicate more social support [28]. Number of previous substance use treatment episodes was also assessed.

Insurance status was abstracted from the medical record. Clinical intake assessments were reviewed to obtain substance use history and treatment characteristics. Opioid use items at intake included: type of opioid, route, frequency, age of first use, and history of overdose. Lifetime polysubstance use excluded tobacco. Length of current treatment episode was the number of days between the date of buprenorphine induction and initial clinic visit date through the survey date. Long-term MOUD treatment with buprenorphine is defined as ≥ 1 year [29].

### Data analysis

Descriptive statistics were generated. Pearson χ2 and Fisher’s exact test were used to test differences by gender for categorical variables; T-tests and Mann–Whitney U test were used to test differences by gender and length of treatment for continuous variables. Multivariate linear regression was conducted to identify a relationship between BARC-10 total score and length of current treatment episode when controlling for gender, race, age, and discrimination. Variables included in the multivariate models as potential confounders in recovery capital were chosen based on existing literature [19, 23, 24] and clinical experience. Additional possible confounding variables could not be included in the model due to small sample size. Significance was set at 0.05 and analyses were performed using SPSS version 26.

## Results

Table 1 presents demographic and psychosocial characteristics for the overall sample (*n* = 130) and by gender. Seventy-one (54.6%) participants were women. Most participants identified as Black, were single, and had at least a high school education. About half were unemployed and over half had public insurance. Women were younger and more likely than men to be unemployed and live alone with children. In the past year, approximately one in three participants experienced homelessness and one in two experienced food insecurity. One-third of participants reported recent discrimination in a healthcare setting due to drug or alcohol use. Many participants had seen a non-addiction healthcare provider at least once in the previous 12 months. One in two participants reported a psychiatric comorbidity. On average, participants reported social support some to most of the time.

**Table 1:** Demographic and psychosocial characteristics of a sample receiving buprenorphine for opioid use disorder

Demographic and psychosocial characteristics	Total	Men	Women	P value
	N (%)	N (%)	N (%)	
	*N* = 130	*N* = 59	*N* = 71	
Age (mean ± SD)	42.4 ± 12.3	47.1 ± 11.9	38.8 ± 11.3	** < .001**
Race				0.227
Black	87 (67.4)	43 (74.1)	44 (62.0)	
White	34 (26.4)	11 (19.0)	23 (32.4)	
Other	8 (6.2)	4 (6.9)	4 (5.6)	
Employment				**0.042**
Employed	33 (25.4)	21 (35.6)	12 (16.9)	
Unemployed	70 (53.8)	26 (44.1)	44 (62.0)	
Receiving disability	27 (20.8)	12 (20.3)	15 (21.1)	
Insurance				0.668
Public	78 (60.0)	34 (57.6)	44 (62.0)	
Private	15 (11.5)	6 (10.2)	9 (12.7)	
None	37 (28.5)	19 (32.2)	18 (25.4)	
Education				0.604
Less than high school education	27 (20.8)	14 (23.7)	13 (18.3)	
High school education	65 (50.0)	30 (50.8)	35 (49.3)	
More than high school education	38 (29.2)	15 (25.4)	23 (32.4)	
Single marital status	89 (68.5)	38 (64.4)	51 (71.8)	0.364
Living arrangement				**0.003**
With sexual partner	37 (29.1)	15 (25.9)	22 (31.9)	
Alone with children	13 (10.2)	2 (3.4)	11 (15.9)	
With family/friends	42 (33.1)	19 (32.8)	23 (33.3)	
Alone	21 (16.5)	17 (29.3)	4 (5.8)	
Other	14 (11.0)	5 (8.6)	9 (13.0)	
Homeless (past 12 months)	48 (37.2)	25 (42.4)	23 (32.9)	0.265
Food insecurity (past 12 months)	73 (57.0)	31 (54.4)	42 (59.2)	0.588
Substance use discrimination in healthcare setting (past 12 months)	37 (29.1)	12 (21.1)	25 (35.7)	0.071
Healthcare access (past 12 months)	114 (87.7)	51 (86.4)	63 (88.7)	0.692
Psychiatric comorbidity	73 (56.2)	29 (49.2)	44 (62.0)	0.143
^a^Social support [Median (IQR)]	3.6 (2.9–4.4)	3.2 (2.4–4.4)	3.9 (3.0–4.4)	0.083

SD, Standard deviation; Mos, months; IQR, interquartile range. Boldface indicates significant at p ≤ .05

^a^Social support scale range 0–5

Substance use history and treatment characteristics are summarized in Table 2. Heroin was the most common type of opioid use, and approximately half of participants reported injection drug use. On average, participants were 24 years of age at the onset of opioid use. Most participants were using illicit or misusing prescription opioids daily at treatment entry and nearly all reported current or past polysubstance use. Men reported more treatment episodes and were more likely to have had a previous overdose than women. Across gender, over half of participants had received long-term MOUD with buprenorphine with the average length of the current treatment episode of 396 (SD = 246) days.

**Table 2:** Substance use history and treatment characteristics of a sample receiving buprenorphine for opioid use disorder

Substance use history and treatment characteristics	Total	Men	Women	*P* value
	*N* (%)	*N* (%)	*N* (%)	
	*N* = 130	*N* = 59	*N* = 71	
Lifetime type of opioid use	90 (69.2)	49 (83.1)	41 (57.7)	**0.005**
Heroin only	14 (10.8)	2 (3.4)	12 (16.9)	
Prescription opioid only	26 (20.0)	8 (13.6)	18 (25.4)	
Both				
Lifetime most serious route of opioid use				**0.04**
Injection	63 (52.9)	31 (57.4)	32 (49.2)	
Nasal	45 (37.8)	22 (40.7)	23 (35.4)	
Oral	11 (9.2)	1 (1.9)	10 (15.4)	
Age onset opioid use	24.36 ± 9.2	23.2 ± 8.5	25.36 ± 9.7	0.203
(Mean ± SD)				
Lifetime history overdose	27 (35.1)	17 (50.0)	10 (23.3)	**0.015**
Lifetime polysubstance use	122 (93.8)	57 (96.6)	65 (91.5)	0.232
# Lifetime treatment episodes [Median (IQR)]	3 [1–4]	3 [1–4]	2 [1–3]	0.053
Daily opioid use at treatment entry (illicit or Rx misuse)	98 (76.6)	48 (82.8)	50 (71.4)	0.132
^a^Long-term MOUD with buprenorphine (≥ 1 year)	74 (56.9)	33 (55.9)	41 (57.7)	0.835
^a^Length of current treatment episode (days; Mean ± SD)	396.1 ± 245.9	385.7 ± 239.3	404.8 ± 252.5	0.662

SD, standard deviation; IQR, interquartile range. Boldface indicates significant at p ≤ .05

^a^At time of survey completion

Recovery capital is summarized in Table 3. Average total BARC-10 score was 44.9 (SD = 9.8). Mean BARC-10 scores did not differ between individuals who had received long-term MOUD and those who were newer to MOUD treatment (44.8 ± 10.6 vs. 45.0 ± 8.8; p = 0.928; data not shown). Overall, average individual BARC-10 item scores ranged from 3.7 to 5.3 with the highest scores on the substance use and sobriety item in the health dimension and the risk-taking item in the purpose dimension (Table 3). The lowest item scores were the psychological health item in the health dimension and the community involvement and social support items within the community dimension. Gender differences are also summarized in Table 3. Specifically, women scored significantly higher than men on the substance use and sobriety, risk-taking, and recovery experience items in the health and purpose dimensions. Within the home and community recovery dimensions, scores did not differ by gender for any individual items.

**Table 3:** BARC-10 individual item and total scores for a sample receiving buprenorphine for opioid use disorder

BARC-10 items within SAMSHA’s recovery dimensions	Total	Men	Women	*P* value
	*N* = 130	*N* = 59	*N* = 71	
	Mean (SD)	Mean (SD)	Mean (SD)	
*Health dimension*				
There are more important things to me in life than using substances	5.3 (1.3)	5.2 (1.3)	5.5 (1.4)	**0.005**
In general, I am happy with my life	4.1 (1.5)	4.0 (1.4)	4.2 (1.5)	0.426
I have enough energy to complete the tasks I set for myself	4.3 (1.4)	4.5 (1.3)	4.2 (1.5)	0.31
*Home dimension*				
My living space has helped to drive my recovery journey	4.3 (1.5)	4.1 (1.5)	4.4 (1.6)	0.15
*Community dimension*				
I am proud of the community I live in and feel a part of it	3.7 (1.7)	3.9 (1.6)	3.5 (1.7)	0.183
I get lots of support from friends	3.9 (1.5)	3.9 (1.5)	4.0 (1.6)	0.625
*Purpose dimension*				
I am happy dealing with a range of professional people	4.8 (1.2)	4.7 (1.1)	5.0 (1.2)	0.051
I regard my life as fulfilling and without the need for using drugs or alcohol	4.3 (1.4)	4.3 (1.4)	4.4 (1.4)	0.486
I take full responsibility for my actions	5.2 (1.2)	5.0 (1.2)	5.4 (1.1)	**0.005**
I am making good progress on my recovery journey	4.9 (1.2)	4.6 (1.2)	5.1 (1.1)	**0.006**
BARC-10 total score	44.9 (9.8)	44.1 (9.6)	45.6 (9.9)	0.17

BARC-10, Brief Assessment of Recovery Capital [10-items]. Boldface indicates significant at p ≤ .05. BARC-10 individual item range 1–6; total score range 10–60. Nonparametric analyses using Mann–Whitney U test

The overall linear regression model, including gender, race, age, discrimination, and length current treatment episode, did not significantly predict BARC-10 total score, [F[5, 119] = 1.58; p = 0.170] and only accounted for 6.2% of the variance (Table 4). Length of current treatment episode was not associated with BARC-10 total score. However, recent discrimination in a healthcare setting was significantly associated with a lower BARC-10 score (p = 0.020).

**Table 4:** Multivariate analysis of association between BARC-10 total score and length of opioid use disorder treatment (*N* = 124)

Factors associated with BARC-10 total score	B (95% CI)	*b*	*t*	*P* value
^a^Length of current treatment episode	0.004 (-0.003, 0.010)	0.1	1.06	0.293
Age	-0.02 (-0.18, 0.13)	-0.03	-0.29	0.773
Gender				
Men (referent)	-	-	-	-
Women	1.99 (-1.50, 5.47)	0.11	1.13	0.261
Race				
White/other (referent)	-	-	-	-
Black	-0.94 (-4.80, 2.93)	-0.05	-0.48	0.633
Recent substance use discrimination in healthcare setting				
No (referent)	-	-	-	-
Yes	-4.31 (-7.94, -0.68)	-0.21	-2.35	**0.02**

OUD, opioid use disorder; BARC-10, Brief Assessment of Recovery Capital. Boldface indicates significant at p ≤ .05

^a^Length of current treatment episode at time of survey completion

## Discussion

The present study found that recovery capital was high for many men and women receiving outpatient MOUD with buprenorphine. While recovery capital did not differ by length of current treatment episode within our clinical sample, recovery capital was not uniform across SAMSHA’s recovery dimensions or gender. Additionally, recent discrimination in a health care setting negatively impacted recovery capital. Strengthening existing recovery capital as well as cultivating new recovery capital may help improve person-centered recovery outcomes, such as quality of life, among individuals receiving MOUD at varying stages of reduced or eliminated substance use.

Surprisingly, total recovery capital among our overall sample was similar to a US sample of nontreatment seeking individuals with a resolved drug or alcohol problem of 12 years, on average [16]. While we hypothesized that length of current treatment episode would be associated with level of recovery capital, the lack of significant finding is likely due to several factors that ultimately contributed to the unexpectedly high level of recovery capital among participants. First, participants may have entered treatment with high levels of recovery capital. Considering the individual and system-level barriers to MOUD treatment, people with greater recovery capital may be more likely to identify and access treatment than those with lower recovery capital [11, 30]. Additionally, the outpatient MOUD treatment setting, as opposed to more intensive SUD treatment, is often a good fit for individuals with high levels of existing recovery capital [31]. Second, our null finding may be due to differential treatment attrition, selection bias, and a limited study sample. For example, individuals with lower levels of recovery capital may have discontinued treatment, transitioned to a higher level of SUD treatment, and/or chosen not to participate in the study. Finally, the BARC-10 was developed and normed in a non-US sample of majority White males and interpreted based on the abstinence only recovery definition [26]. Therefore, it may not have comprehensively captured differences in recovery capital from a contemporary, holistic perspective among our clinical sample of majority female and Black participants. Nonetheless, our finding that MOUD participants had similar levels of recovery capital as people in remission from SUD in the non-treatment population highlights how MOUD can provide a strong platform for people with OUD to maintain recovery [32] despite the stigma surrounding MOUD [33] that leads some to perceive MOUD as not compatible with recovery.

Across the recovery dimensions, one of the highest scored recovery capital items was the substance use and sobriety item within the health recovery dimension. This is consistent with literature supporting the efficacy of MOUD with buprenorphine to reduce the effects of physical opioid dependence, including craving and withdrawal symptoms, and help patients maintain abstinence [34]. Although our sample had access to additional multimodal treatment services (e.g., medical care, mental health counseling, case management) that are commonly available with opioid-based outpatient treatment centers, we still found some dimensions of recovery capital, including psychological health and community, to need further improvement. Among individuals with OUD, psychiatric comorbidities are prevalent and associated with negative treatment outcomes, including increased overdose risk [35]. However, when utilized, adjunctive personalized psychosocial interventions can improve MOUD outcomes [36]. Additionally, having a positive social network and a sense of community belonging improves treatment and recovery outcomes [37]. Importantly, the success of efforts to strengthen community support for people in OUD treatment can be limited by stigma surrounding OUD and OUD medications [33]. Thus, incorporating treatment adjuncts focused on overcoming stigma, such as engaging patients’ partners and families in OUD treatment, may improve recovery capital for some patients. Overall, recovery capital may be a promising alternative treatment target to abstinence as targeting recovery capital in treatment may improve resources and support networks that help reduce risk even with substance use recurrence. Integrating recovery capital assessments into MOUD could be useful to systematically tailor available multimodal services to an individual’s specific recovery needs while simultaneously integrating harm reduction principles into addiction treatment.

In addition to differences across recovery dimensions, gender-based differences in recovery capital emerged within the health and purpose recovery dimensions highlighting the importance of incorporating gender-informed approaches in recovery capital measurement and intervention. Prior work among nontreatment seeking populations highlighted women having more compromised long-term recovery dimensions (i.e., quality of life and psychological distress) than men [16]. Conversely, the current study found women were more likely than men to report having more important things in their lives than substances, making good progress on their recovery journey, and taking responsibility for their actions. These heterogeneous findings may be reflective of the conflicting impacts women’s unique responsibilities, such as often serving as the primary caregiver for children, can have on recovery progress. Additionally, these mixed findings may be due, in part, to differences in study populations. For example, sex differences have been found for OUD treatment outcomes specific to MOUD with buprenorphine, indicating a possible female advantage [38]. More work is needed to understand the role of sex and gender in recovery. Further, tailored interventions for men across recovery dimensions are warranted given men reported lower recovery capital in health and purpose recovery dimensions as well as higher disease severity compared to women.

Nearly half of participants reported recent discrimination in a healthcare setting which was associated with compromised recovery capital, similar to findings from a previous pilot study among individuals in SUD recovery [19]. Discrimination could have detrimental impacts on maintenance and accrual of recovery capital that in turn negatively affect long-term recovery outcomes such as treatment engagement. Unfortunately, stigma toward individuals with OUD is prevalent within the healthcare community [39], and women are especially vulnerable to stigma and discrimination for having a SUD [40]. Therefore, OUD education efforts and stigma-reduction strategies should include healthcare providers [41]. Decreasing stigma in healthcare may help improve overall health and support long-term recovery of people with OUD.

There are several limitations to this study. First, the cross-sectional design and small sample size of the present study limited our ability to evaluate recovery capital trajectories throughout treatment and differentiate between existing recovery capital at treatment entry and recovery capital obtained during treatment. Additionally, participants self-selected into this convenience sample from one outpatient substance use treatment clinic limiting the generalizability of our results and potentially limiting variability in recovery capital scores as individuals with lower recovery capital may have been more likely to discontinue treatment or chosen not to participate. Information bias is probable for the variables obtained from our chart review. Our small sample size may have impacted the ability to detect statistically significant differences. Gender identity was assessed in the survey, but no participants identified as a gender minority. Thus, our analyses were limited to a cisgender conceptualization of gender identity. Lastly, our recovery capital measure was limited to the BARC-10 rather than the full 50-item ARC or other recovery capital measures due to the nature of secondary data analysis.

Despite these limitations, to our knowledge, this study is the first to describe recovery capital among men and women receiving outpatient MOUD with buprenorphine. Future research can build upon the current study by evaluating gender-stratified recovery capital trajectories throughout MOUD treatment to differentiate between existing recovery capital at treatment entry and recovery capital obtained during treatment. Further, gender-informed, clinically feasible recovery capital measures need to be developed and validated specifically for this population. Longitudinal recovery capital studies incorporating measures of psychosocial functioning, risk reduction strategies, and substance use are needed to better understand how targeting recovery capital may be used as a harm reduction intervention in SUD treatment settings. Additionally, findings suggest harm reduction may have a role to play in supporting and enhancing recovery capital for people prior to treatment entry and in the interstices between treatments. Studies among these populations are needed to investigate this further. Lastly, hypothesis-driven longitudinal studies should be conducted to investigate the relationship between discrimination and recovery capital.

## Conclusions

Regardless of length of time in treatment, most individuals receiving outpatient MOUD with buprenorphine reported high overall recovery capital suggesting they have resources to improve their health and wellness, live a self-directed life, and strive to reach their full potential. However, recovery capital varied across recovery dimensions as well as by gender, and many participants experienced recent discrimination in a healthcare setting which can have a detrimental impact on recovery capital. Findings highlight areas for tailored treatment interventions to strengthen and cultivate recovery capital. Targeting recovery capital as an alternative person-centered treatment outcome to abstinence could serve as a beneficial harm reduction strategy and help promote recovery-based systems of care for people with OUD.

## Data Availability

The datasets generated and analyzed during the current study are available from the corresponding author on reasonable request.

## Abbreviations

SUD: Substance use disorder
MOUD: Medication for opioid use disorder
SAMHSA: Substance abuse and mental health services administration
OUD: Opioid use disorder
BARC-10: Brief assessment of recovery capital
ARC: Assessment of recovery capital
US: United States
